# Structure analysis of free and bound states of an RNA aptamer against ribosomal protein S8 from *Bacillus anthracis*

**DOI:** 10.1093/nar/gku743

**Published:** 2014-08-19

**Authors:** Milya Davlieva, James Donarski, Jiachen Wang, Yousif Shamoo, Edward P. Nikonowicz

**Affiliations:** 1 Department of Biochemistry and Cell Biology, Rice University, Houston, TX 77251–1892, USA; 2 Food and Environment Research Agency, Sand Hutton, York, YO41 1LZ, United Kingdom; 3 Department of Physics, East China Normal University, 200062 Shanghai, P. R. China

## Abstract

Several protein-targeted RNA aptamers have been identified for a variety of applications and although the affinities of numerous protein-aptamer complexes have been determined, the structural details of these complexes have not been widely explored. We examined the structural accommodation of an RNA aptamer that binds bacterial r-protein S8. The core of the primary binding site for S8 on helix 21 of 16S rRNA contains a pair of conserved base triples that mold the sugar-phosphate backbone to S8. The aptamer, which does not contain the conserved sequence motif, is specific for the rRNA binding site of S8. The protein-free RNA aptamer adopts a helical structure with multiple non-canonical base pairs. Surprisingly, binding of S8 leads to a dramatic change in the RNA conformation that restores the signature S8 recognition fold through a novel combination of nucleobase interactions. Nucleotides within the non-canonical core rearrange to create a G-(G-C) triple and a U-(A-U)-U quartet. Although native-like S8-RNA interactions are present in the aptamer-S8 complex, the topology of the aptamer RNA differs from that of the helix 21-S8 complex. This is the first example of an RNA aptamer that adopts substantially different secondary structures in the free and protein-bound states and highlights the remarkable plasticity of RNA secondary structure.

## INTRODUCTION

Over the past several years, high-resolution structure studies of ribonucleoprotein complexes have revealed a wealth of detailed information on structural motifs that contribute to protein–RNA specificity (1–5). RNA molecules employ a diverse repertoire of secondary structure motifs including bulged nucleotides, non-canonical base pairs and base triples, terminal (hairpin) loops and internal loops to create architectures that serve as protein-specific conformational signatures. Internal loops, regions of double-stranded nucleic acid within a base-paired helix that do not maintain Watson–Crick secondary structure, occur in a variety of RNA systems and widely differ in their size and nucleotide content (6). Hydrogen bonding, base stacking and divalent metal ion coordination can stabilize complex folds of these regions, but with a few notable exceptions such as the loop E motif, kink-turns, tetra-loops, C-loops and the A-minor motif, it remains difficult to predict the interactions that form among the internal loop nucleotides in free or protein-bound forms of an RNA (6,7).

**Figure 1.**
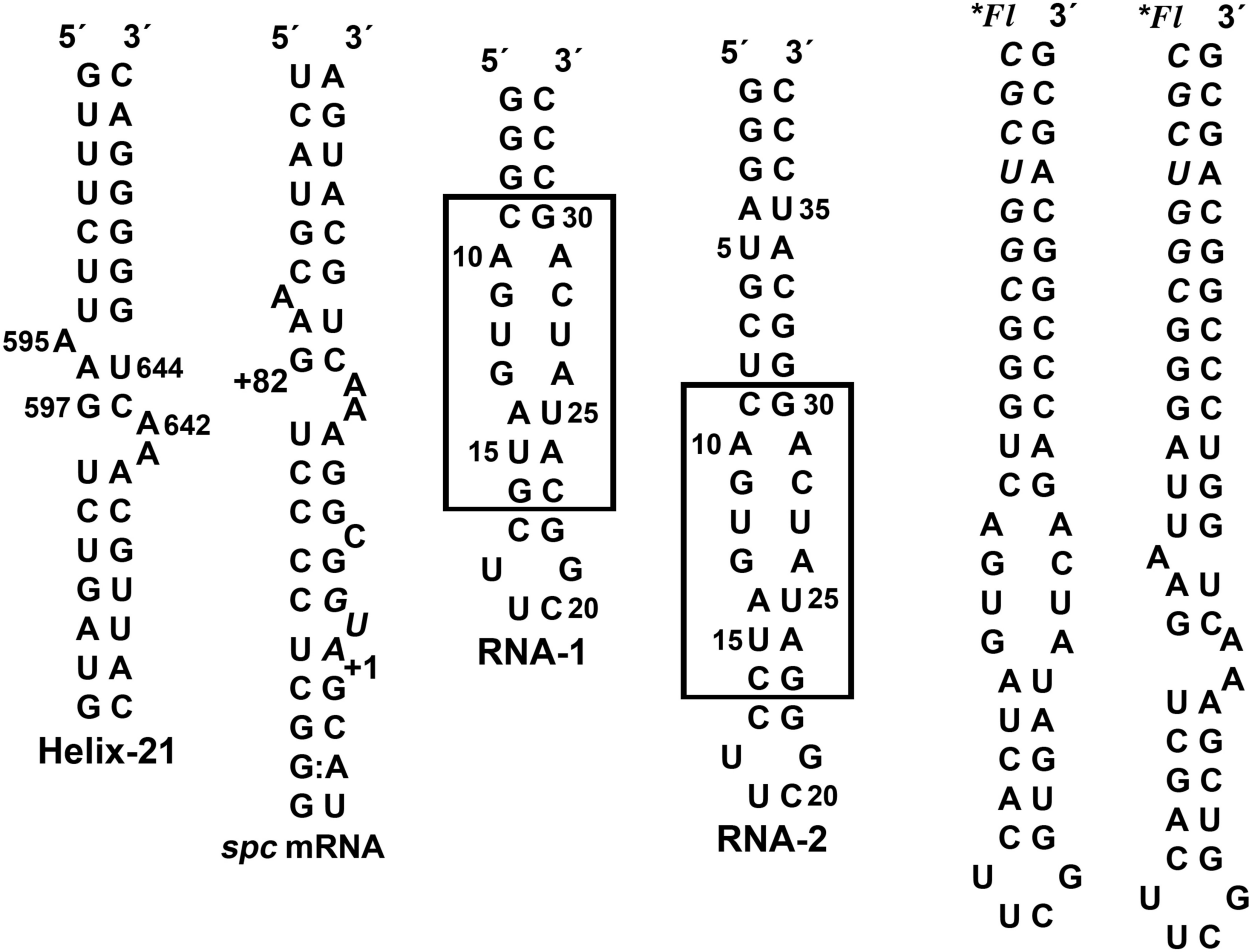
Sequence and secondary structures of primary RNA binding sites for r-protein S8. The natural binding sites are helix 21 from *Bacillus* 16S rRNA and the *spc* mRNA from *E. coli*. The RNA aptamer constructs used for the structural studies are RNA-1 (NMR) and RNA-2 (X-ray) and the randomized element from the selection is boxed. 5′-fluorescein-labeled RNA hairpins were prepared by ligation of a chemically synthesized oligonucleotide (*italics*) with enzymatically synthesized oligonucleotides.

The complex formed between bacterial ribosomal protein S8 (r-protein S8) and 16S rRNA is a well-studied interaction that is specified by an internal loop. The binding of r-protein S8 to 16S rRNA has been extensively characterized using a variety of techniques including chemical modification and protection assays (8–10), filter binding assays (11–13) and mutagenesis (13,14). These studies showed that the majority of protein–RNA contacts localize to helices 21 and 25 and that a minimal RNA fragment located in helix 21 is sufficient to confer specificity and high affinity to the S8-RNA interaction (12). This primary binding site consists of a helix interrupted by an internal loop of seven phylogenetically conserved nucleotides (Figure 1). The same conserved secondary structure element is found in the 5′ untranslated region of the *rplE* gene at the beginning of the *spc* operon (15). The translation of genes encoded by the *spc* operon, including those of S8 and several other ribosomal proteins, is repressed by the binding of r-protein S8 at this site (15).

In addition to structural conservation of the primary RNA binding sites for S8, the overall fold of S8 r-proteins is conserved. The S8 protein has two domains, N- and C-terminal (16–18), and the arrangement of α-helices and β-sheet strands that make up the domains is maintained when S8 binds to RNA (19–21). In addition, many of the intermolecular interactions between r-protein S8 and RNA are the same for helix 21 and the *spc* mRNA binding sites. Notably, these protein–RNA interactions are maintained within the 30S ribosomal RNA subunit. These complexes reveal that the S8-RNA binding involves electrostatic and hydrogen-bond interactions and shape complementarity.

The primary RNA binding site for r-protein S8 contains non-canonical structural elements important for specificity and affinity. A previous systematic evolution of ligands by e*x*ponential enrichment (SELEX) study suggested the presence of a base triple (G_597_-C_643_)-U_641_ located in the internal loop of helix 21 (22). The RNA molecules that bound tightly to the S8 protein contained nucleotide combinations at the positions corresponding to 597/643/641 that were isosteric with the proposed (G-C)-U base triple. In addition to the base triple, an adenine nucleotide corresponding to the invariant A_642_ was present in the selected aptamers (22), underscoring the importance of this residue. In the free RNA, U_641_ participates in a bifurcated hydrogen bond with the G_597_-C_643_ base pair and the A_642_ base (23). The internal loop also contains the base triple A_595_-(A_596_-U_644_) (23). These elements are important for shaping the trajectory of the sugar-phosphate backbone to display a distinctive set of structural features and are preserved in S8-RNA complexes (19–21,24). A complementary study involving the randomization of residues 597/641/643 and performed *in vivo* using *Escherichia coli* confirmed the functional importance of the nucleotide triplet (25).

The nucleotide sequence and secondary structure of the primary binding site for r-protein S8 on helix 21 are highly conserved (Figure 1B). These conserved elements impose a shape to the RNA that optimizes electrostatic and van der Waal's interactions with the protein surface. The S8 protein contains a secondary RNA binding site with a large electropositive surface associated with helix 25 in the 30S subunit but does not display sequence specificity. To search for RNA secondary structures that differ from the conserved bacterial motif and investigate how RNA sequence might adapt to binding restrictions imposed by the S8 protein, a SELEX experiment was performed. The selection was based on an RNA stem-loop scaffold containing symmetric and asymmetric internal loops of 16 randomized nucleotides. An RNA aptamer sequence that is not predicted to adopt the structural features of the highly conserved asymmetric internal loop motif of the natural binding site was chosen for structure analysis. The affinity of the aptamer for the S8 protein is 2-fold tighter than the affinity exhibited by the native helix 21. In the free state, the internal loop of the hairpin stem contains G-A, U-U and A-A mismatches with an overall helical A-form geometry. To bind r-protein S8, the internal loop undergoes a dramatic rearrangement of secondary structure to form a base triple and a base quartet. Many of the protein contacts observed in native S8-RNA complexes are now recapitulated in a novel manner in the S8-aptamer complex. It is remarkable that a molecule whose secondary structure is far removed from the native target forms many of the same contacts as the natural binding site. This is the first example of an RNA aptamer shown to have one dominant secondary structure in the free state and a substantially different structure in the protein-bound state. The S8-aptamer complex demonstrates the remarkable plasticity of RNA to form unexpected structures that meet biological function.

## MATERIALS AND METHODS

### Protein expression and purification

The *Bacillus anthracis* and *E. coli* S8 r-proteins were expressed as N-terminal 6X-His tagged fusion proteins. The *rpsH* genes were polymerase-chain-reaction-amplified from genomic DNA, cloned into the pET28b vector (Novagen) using the Nhe1-Xho1 restriction sites, and the sequences confirmed. The proteins were expressed in BL21(DE3) cells, isolated in the form of inclusion bodies, and dissolved with 7 M urea as described (13). The *B. anthracis* S8 r-protein also was expressed from cells cultured in M9 media supplemented with 50 mg/l selenomethionine. The urea-solubilized S8 r-protein solutions were applied to an affinity (Ni^2+^) column that was equilibrated with buffer A (7 M urea, 0.1 M NaH_2_PO_4_, 10mM tris-HCl, pH 8.0 and 2 mM β-mercaptoethanol). The column was washed with buffer A plus 20 mM imidazole and 500 mM NaCl and the protein was eluted using 200 mM imidazole in buffer A. Fractions were collected and those containing S8 (>95% purity) were combined and the protein renaturated over 3 days via serial dialysis in buffer B (50 mM KCl, 20 mM sodium cacodylate, pH 6.8) containing decreasing molar concentrations of urea: 7.0, 4.0, 2.0, 1.0, 0.5 and 2× 0.0 M. The refolded S8 proteins were concentrated (Amicon) and quantified using the Bradford method.

### *In vitro* aptamer selection

The RNA aptamer selection was performed as described (26–28) using 5′-rNTPs. The RNA transcript was designed to form a hairpin with the sequence 5′-GAGGCUUCCU(N*_X_*)CUUCGG(N_*Y*_)GGGAAGCCUC-3′. *X* and *Y* designate the number of randomized nucleotides (*X* = 7, 8, 9 and *Y* = 9, 8, 7) so that the sum of *X* and *Y* was fixed at 16. The aptamer sequence chosen for study was identified after 10 rounds of selection and forms a secondary structure with a symmetric internal loop, in contrast to the asymmetric internal found in natural S8 binding sites. Additional details of the selection are given in *Supplementary Information*.

## Preparation of RNA samples

The aptamer molecule for X-ray crystallography (Figure 1) was purchased (Dharmacon). The aptamer molecules for NMR (Figure 1) were synthesized *in vitro* using T7 RNA polymerase and a synthetic DNA template. Unlabeled and isotopically labeled RNA molecules were prepared as described (29). The polyacrylamide gel electrophoresis (PAGE) purified RNA molecules were dialyzed extensively against 10 mM KCl, 10 mM sodium cacodylate, pH 6.6 and 0.02 mM EDTA and lyophilized. The RNA samples were suspended in 0.35 ml of 99.96% D_2_O or 90% H_2_O/10% D_2_O and annealed and contained 30–140 A_260_ OD units of RNA oligonucleotide (≈0.4–1.5 mM). For fluorescence anisotropy experiments, 5′-fluorescein-labeled RNA hairpins were prepared by ligation of a 5′-fluoroscein-labeled RNA heptamer (Dharmacon) with *in vitro* transcribed RNA sequences corresponding to the aptamer or the r-protein S8 binding site on helix 21. The labeled hairpins were PAGE purified, dialysed against 150 mM KCl and 10 mM sodium cacodylate, pH 6.6, and stored at −80°C.

### NMR spectroscopy and structure determination of the RNA aptamer

Spectra were acquired on Varian Inova 500 MHz (^1^H-[^13^C, ^15^N, ^31^P] probe) and 600 MHz and 800 MHz (^1^H-[^13^C, ^15^N] cryoprobe) spectrometers and NMR spectra were processed and analyzed using Felix 2007 (Felix NMR Inc., San Diego, CA).

Two-dimensional (2D) ^13^C-^1^H HSQC spectra were collected to identify ^13^C-^1^H chemical shift correlations. Sugar spin systems were assigned using 3D HCCH-TOCSY (8 ms and 24 ms DIPSI-3 spin lock) experiments and 2D HCN experiments were used to identify intra-residue base-ribose correlations. Pyrimidine C2 and C4 resonances were assigned from H6-C2 and H5-C4 correlations using 2D H(CN)C and 2D CCH-COSY experiments and a 2D H(N)CO experiment for uridine NH-[C2, C4] resonances (30–32). Sequential assignments and distance constraints for the non-exchangeable resonances were derived at 26°C from 2D ^1^H-^1^H NOESY spectra (*t*_m_ = 90, 180 and 320 ms) and 3D ^13^C-edited NOESY spectra (*t*_m_ = 180 and 360 ms). Assignments and distance constraints for the exchangeable resonances were derived at 12°C from 2D NOESY spectra (*t*_m_ = 160 and 360 ms) acquired in 90% ^1^H_2_O. ^3^J_H-H_, ^3^J_P-H_ and ^3^J_C-P_ coupling constants were estimated using DQF-COSY, ^31^P-^1^H HetCor and CECT-HCP (33) experiments, respectively. NOE peak intensities were classified as very strong, strong, medium, weak, or very weak and distance constraints applied (Supplementary Table S1).

Structure refinement was carried out with simulated annealing and restrained molecular dynamics (rMD) calculations using Xplor-NIH v2.19 (34). The aptamer was generated as a linear molecule and starting coordinates were based on A-form geometry. Beginning with the energy minimized starting coordinates, 50 structures were generated using 18 ps of rMD at 1200 K with hydrogen bond, NOE-derived distance and base-pairing restraints. The system then was cooled to 25 K over 12 ps of rMD. Force constants used for the calculations were increased from 2 kcal mol^−1^ Å^−2^ to 30 kcal mol^−1^ Å^−2^ for the NOE and from 2 kcal mol^−1^ rad^−2^ to 30 kcal mol^−1^ rad^−2^ for the dihedral angle constraints. After minimization, NOESY spectra were re-examined for predicted NOEs absent from the constraint list. The calculations were repeated using revised constraint lists and eight structures were selected for the final refinement using criteria that included lowest energies, fewest constraint violations and fewest predicted unobserved NOEs. A second round of rMD was performed on these structures using a starting temperature of 300 K followed by cooling to 25 K over 28 ps of rMD. The eight refined structures were analyzed using Xplor-NIH and Chimera. The data and structure statistics are reported in Supplementary Table S1.

### Crystallization and structure of *B. anthracis* S8 and *B. anthracis* S8-aptamer complex

Crystals of *B. anthracis* S8 were obtained by sparse matrix screening of S8 at 10 mg/ml at 4ºC. Preliminary results were followed by optimization of the successful condition manually using the sitting drop vapor diffusion method. The best-quality crystals were grown in 48–51% Tacsimate at 20ºC. No cryoprotectants were required for cryopreservation in liquid nitrogen.

S8-aptamer complexes were formed by mixing RNA aptamer (5 mM MgCl_2_, 75 mM KCl, 2 mM DDT, 20 mM MOPS pH 7.0) and S8 protein (20 mM cacodylic acid pH 6.3, 100 mM KCl, 5 mM BME) in a 1:1 mole ratio to a final concentration of 150 μM followed by incubation for 1 h on ice. The final crystallization condition was 0.3 M di-ammonium hydrogen citrate, 100 mM sodium chloride, 16% PEG 3350 and 10 mM spermidine at 10°C.

### Data collection and processing

Diffraction data sets for S8 protein were collected at 100 K at 1.9 Å at the Cornell High Energy Synchrotron Source beam line. The data were integrated, scaled and merged using the HKL-2000 package (35). *B. anthracis* r-protein S8 crystallized in space group C2221 with the unit cell parameters *a* = 118.33, *b* = 148.82, *c* = 68.62 Å, α = γ = 90°, β = 98.7°. Data collection and processing statistics are listed in Supplementary Table S2.

Crystals of S8-RNA were passed briefly through cryoprotectant solutions consisting of 0.3 M sodium citrate pH 7.0, 100 mM sodium chloride, 10 mM spermidine supplemented with 5, 10, 15, or 20% (v/v) glycerol. Diffraction data for the S8-aptamer complex was collected to 2.6 Å resolution using a NOIR-1 Molecular Biology Consortium (MBC) detector system at the beamline 4.2.2 at the Advanced Light Source synchrotron (Berkeley, CA). The crystal belonged to space group P212121 with unit cell parameters *a* = 55.41, *b* = 59.27, *c* = 92.25 Å, α = β = γ = 90ºC. The data was processed using D*TREK (36) with *R*_merge_ = 9.2% and completeness 99.9%. *R*_merge_ and completeness in the outermost shell (64.3 Å) was 99.9%.

### Structure determination

The structure of *B.* *anthracis* S8 was solved by the standard method of single anomalous dispersion (SAD). Heavy atom sites from the metabolically incorporated selenomethionines were found by the online application SHARP (37). SAD electron density map was calculated using CCP4 (38) and map integration and model building were performed with the program O (39). Molecular replacement for three copies in the asymmetric unit, refinement and composite omit maps was computed using CNS (40). The model was then rebuilt manually and further refined. The final structure has an *R* factor of 22.3% and *R*_free_ of 23.3%.

A molecular replacement for the S8-aptamer complex was found using program Phaser for MR (41) from CCP4 (38) suit using the *B. anthracis* S8 r-protein (solved in-house) as a search model. The initial solution suggested one monomer per asymmetric unit consistent with the Matthew's coefficient of 3.16 (65% of solvent). The molecular replacement was further confirmed by the initial (2F_o_-F_c_) map generated using Coot (42) that clearly indicated electron density for the RNA aptamer not included in the original search model. The S8-RNA model has been refined to the *R* of 18.9% and *R*_free_ 27.0% (Supplementary Table S2). Ramachandran plots and root-mean-square deviations (rmsd) from ideality for bond angles and lengths for S8/RNA were determined using a structure validation program, MolProbity (43).

### Fluorescence anisotropy

A Beacon 2000 fluorescence polarimeter (PanVera Corp.) was used for the fluorescence anisotropy experiments. 5′-fluorescein-labeled RNA hairpin samples were extensively dialyzed against a buffer of 25 mM Tris-Acetate (pH 7.6) and 150 mM potassium acetate. RNA samples were heated to 90°C for 60 s, snap cooled on ice and dialyzed against 25 mM Tris-Acetate (pH 7.6), 150 mM potassium acetate and 10 mM magnesium acetate. The concentration of RNA was kept constant at 1.0 nM and the concentration of S8 protein ranged 1.0–500 nM. Samples were mixed by addition of protein solution to RNA and incubated at 4°C for 30 min. Four measurements were averaged for each S8 concentration. Experiments were performed in triplicate. The apparent *K*_d_ values were determined from a non-linear least-squares fit of the data to a binding model for a single-site using GraphPad Prism 5 (GraphPad Software, Inc.).

## RESULTS

The SELEX experiment was performed to identify RNA sequences that do not maintain the conserved features of helix 21 (Figure 1) but retain the ability to bind the S8 protein with high affinity and specificity. The starting library was composed of molecules with 16 randomized nucleotide positions inserted within the stem of an RNA hairpin (Figure 1C). After 10 rounds of selection, the RNA pool was cloned and 40 inserts sequenced. Alignment of the RNA aptamer sequences showed the presence of native-like (asymmetric internal loop) binding sites including sequences corresponding to helix 21 of *E. coli* and *Bacillus* 16S rRNA in addition to non-natural binding sites with symmetric internal loops. Electrophoretic mobility shift assays (EMSAs) were used to qualitatively assess the S8 binding of non native-like aptamers and the sequence (Figure 1) containing a symmetric internal loop chosen for this study.

Fluorescence anisotropy was used to measure the interaction affinity of the aptamer RNA with r-protein S8 from *Bacillus* and *E. coli*. Fluorescein-labeled RNA aptamer and an RNA molecule corresponding to the primary binding site on helix 21 were titrated with S8 protein and the change in anisotropy of the RNA monitored (Supplementary Figure S1). The *Bacillus* S8 protein binds the RNA aptamer with a *K*_d_ of 110 ± 30 nM and the helix 21 site with a *K*_d_ of 180 ± 60 nM. The affinity of *E. coli* r-protein S8 for the RNA molecules are 8–10-fold tighter, *K*_d_ = 19 ± 4 nM and *K*_d_ = 28 ± 7 nM for the aptamer RNA and helix 21 RNA, respectively. The affinity of *E. coli* r-protein S8 for the helix 21 sequence element is consistent with filter-binding measurements (9,10,12). Filter binding experiments using the archaeal *Methanococcus vannielii* S8 protein yielded an apparent *K*_d_ for its 16S rRNA helix 21 binding site of 220 nM, an affinity similar to that of the *Bacillus* S8 protein for helix 21 (44). S8 proteins from thermophilic and hyperthermophilic archael organisms show 10- to 100-fold tighter binding to their respective 16S rRNA targets (17,44). The affinity of *Aquifex aeolicus* S8 protein for the minimal RNA binding site is 1.5 nM, but the protein has very high affinity (0.018 nM) for an RNA construct containing the three-way junction formed by Helices-20–21–22 (17).

### Solution NMR resonance assignments of the aptamer molecule

The core of the aptamer sequence for NMR analysis was introduced into a hairpin capped by a UUCG tetraloop (Figure 1). Cross peaks in the NH ^15^N-^1^H HSQC spectrum are consistent with the predicted secondary structure including the signature peak at 9.80 ppm from the UUCG tetraloop. Since the selection was performed in the presence of Mg^2+^, the NMR spectrum of RNA-1 was monitored to assess metal ion binding, but only the G-C base pair triplet at the end of the stem exhibited significant metal ion association. Therefore, the solution NMR study of the RNA aptamer was performed in the absence of multivalent cations.

**Figure 2.**
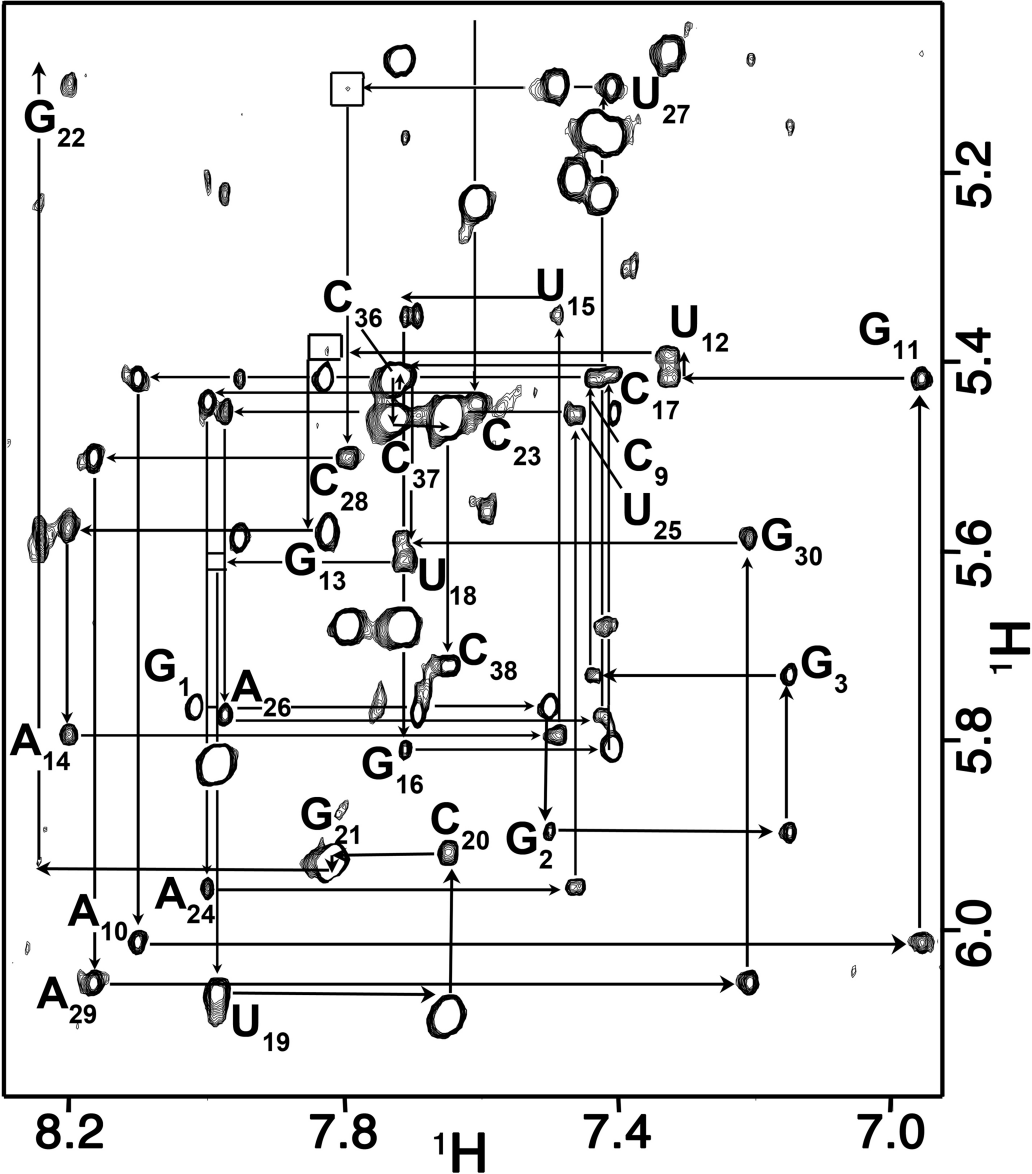
Sequential connectivities through the base-1′ region of the 180 ms mixing time 2D NOE spectrum. The sequential connectivity is very weak between steps U_12_-G_13_ and U_27_-C_28_ (box). The H1′ resonance of G_22_ is shifted upfield to 4.42 ppm. This chemical shift is characteristic of the guanine of a UNCG tetraloop.

Sequential assignments for the non-exchangeable resonances were made using 2D NOESY and 3D ^13^C-edited NOESY experiments. The sequential base-1′ NOE connectivities at τ_m_ = 180 ms (Figure 2) are discontinuous between nucleotides U_18_ and U_19_ of the tetraloop and very weak at steps U_12_-G_13_ and U_27_-C_28_ within the internal loop. The loss of connectivity in the tetraloop is characteristic of the UUCG sequence. Most sequential base 6/8 NOEs are observed except for A_10_-G_11_, U_12_-G_13_ and G_13_-A_14_ in the internal loop. Notably, none of the resonance pairs exhibit exchange broadening (Figure 2 and Supplementary Figure S2) and the nucleotides in the tetraloop are the only residues with ribose resonances that have anomalous chemical shifts. The inter-base pair NOE connectivities of the NH resonances are continuous from G_2_ to G_30_ and from U_15_ to G_21_. The U_12_ and U_27_ NH resonances are at 11.08 and 10.53 ppm, and the NH resonances of G_11_ and U_25_ are not observed. All cytidine NH_2_ resonances were assigned including those of C_28_ (8.01 and 7.04 ppm), which are indicative of base pairing. The inter-nucleotide phosphate ^31^P resonances are clustered between −3.32 and −5.05 ppm except the U_27_pC_28_^31^P resonance that has a chemical shift of −2.40 ppm. A complete list of resonance assignments is given in Supplementary Table S2.

### Solution structure of the RNA Aptamer

**Figure 3.**
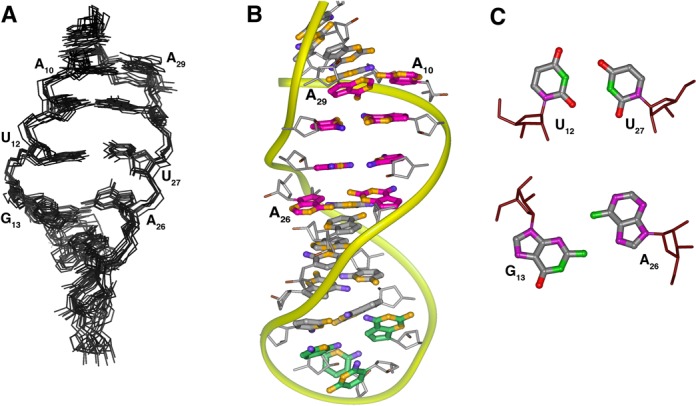
**(A)** Overlay of eight converged solution structures of the RNA-1 aptamer (residues G_3_-G_16_, C_23_-C_36_ shown) and **(B)** average solution structure of the RNA-1 aptamer. The structure calculation used a total of 275 conformationally restrictive distance constraints and 142 dihedral angle constraints (Supplementary Table S2) and the heavy atoms superimpose on the average structure with an average rmsd of 1.54 Å^2^. The color scheme is: magenta, residues in the non-canonical core (A_10_-G_13_ and A_26_-A_29_); green, the tetraloop nucleotides (U_18_-G_21_), orange, nitrogen atoms; blue, oxygen atoms. **(C)** Arrangement of the U_12_-U_27_ and G_13_-A_26_ non-canonical pairs present in the aptamer core.

The global fold the aptamer is a hairpin capped by a canonical UUCG tetraloop and the stem interrupted by an eight-nucleotide internal loop (Figure 3). The internal loop is composed of nucleotides A_10_-G_13_ and A_26_-A_29_ and is flanked on one side by a distorted A_14_-U_25_ base pair. The internal loop is characterized by two non-standard base pairs, a sheared A-G and a U-U, and a Watson–Crick G-C base pair. The bases of A_10_ and A_29_ form an inter-strand stack with each other. The spectral data support the presence of these base–base interactions, but the arrangement of nucleotides is not as tightly ordered as observed in other structures. The H1′ resonance of U_27_ has a chemical shift of 5.03 ppm and is consistent with a partially sheared base pair configuration between G_13_ and A_26_ (31). The U_12_ and U_27_ residues that are adjacent to the G_13_-A_26_ pair form an asymmetric U-U base pair. The U_12_-U_27_ base pair is arranged with the hydrogen-bond pattern U_7_ N3H-U_22_ O4 and U_22_N3H-U_7_ O2 (Supplementary Figure S2). As with the neighboring G_13_-A_26_ pair, the gap between the uridine bases is relatively wide and the imino protons are accessible for solvent exchange. Residue C_28_ pairs with G_11_ as indicated by the NH_2_ and C2 resonances of C_23_, but the G NH resonance exchanges with solvent and is not observed. The A_10_ and A_29_ bases each extend across the helix axis with A_24_ stacked on the G_11_-C_28_ base pair. This conformation is supported by unusually strong cross-strand H2-H1′ NOE cross peaks. The A_10_ base is laterally displaced toward the minor groove and is positioned slightly below the plane of the C_9_-G_30_ base pair. In the converged structures, the A_10_ NH_2_ consistently forms a hydrogen bond with the C_9_ O2. The moderately downfield-shifted A_10_ N6 resonance (82.5 ppm) is consistent with this hydrogen bond.

The sugar-phosphate backbone conformations of the aptamer nucleotides within the internal loop are surprisingly uniform (Figure 3). Only the G_13_ ribose has a C2′-*endo* ring pucker conformation and the uniformly small (<5 Hz) P–C2′ coupling constants for the loop residues place the ϵ torsion angles in the *trans* conformation characteristic of A-form RNA. Although torsion angles α and ζ were left unconstrained, the ζ angle between U_27_ and C_28_ is *trans*-like rather than *gauche^−^* in all structures. This configuration is consistent with the relative downfield ^31^P shift of the involved phosphate. α and ζ at other positions are consistently *gauche^−^* or exhibit *trans/gauche^−^* variability between converged structures.

### Crystal structure of the aptamer-S8 complex

The crystal structure of the aptamer RNA-2 (Figure 1) in complex with *Bacillus* ribosomal protein S8 was solved by molecular replacement using the structure of unliganded *B. anthracis* S8 and refined against a 2.6 Å data set. The refined model contains all 38 nucleotides of the aptamer and residues 4–132 of the S8 protein.

**Figure 4.**
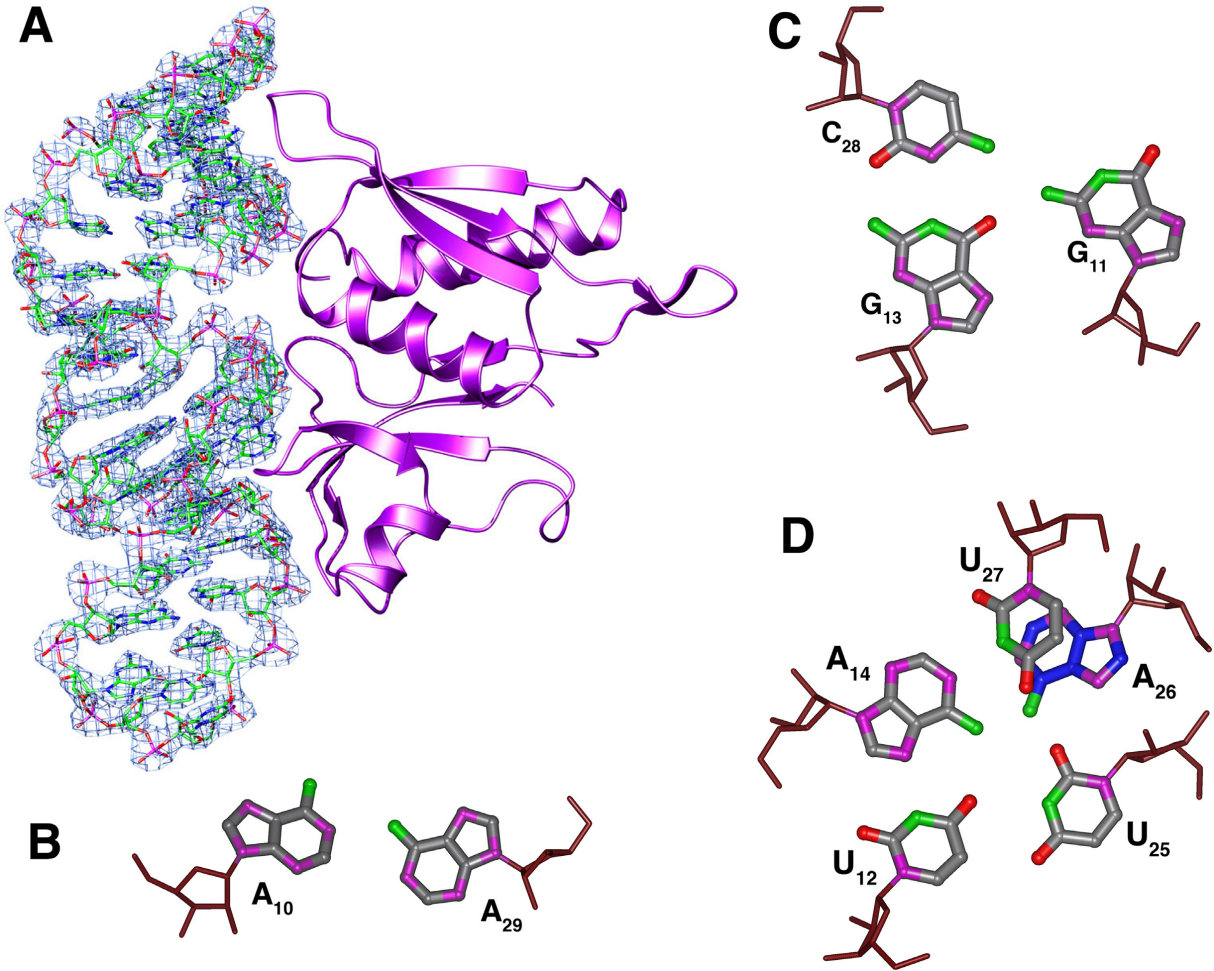
**(A)** The (2mFo-DFc) electron density map countered at 0.4 absolute value of electrons/Å^3^ showing a schematic drawing of *B. anthracis* S8 protein with bound RNA-aptamer. **(B–D)** Non-canonical base-base interactions in the aptamer core. The arrangement of the G_11_-(G_13_-C_28_) base triple is isosteric with the base triple present in the native S8 RNA binding site of helix 21, A_595_-(A_596_-U_644_), but the register of the corresponding residues is different. In the complex, the A_14_-U_25_ base pair is broken and replaced by the A_14_-U_27_ base pair.

The S8 protein has two closely packed domains that are composed of the N- and C-terminal halves of the molecule (Figure 4). The N-terminal domain is made up of an α-β-α-β-β fold and the α-helices stack on the surface of the β-strands. The C-terminal domain contains a short (six residue) α-helix pressed against an anti-parallel four-strand β-sheet. A fifth strand perpendicular to the helix and β-sheet connects these two elements. The structure of the aptamer-bound protein is very similar to the free protein (0.65 Å rmsd of the back bone atoms). The majority of residues that contact the aptamer are in the C-terminal domain of S8 and are generally located in turns at the ends of the β strands. The distribution and arrangement of these secondary structure elements is largely the same as reported for other S8 proteins from thermophilic and mesophilic bacteria (16–21).

**Figure 5.**
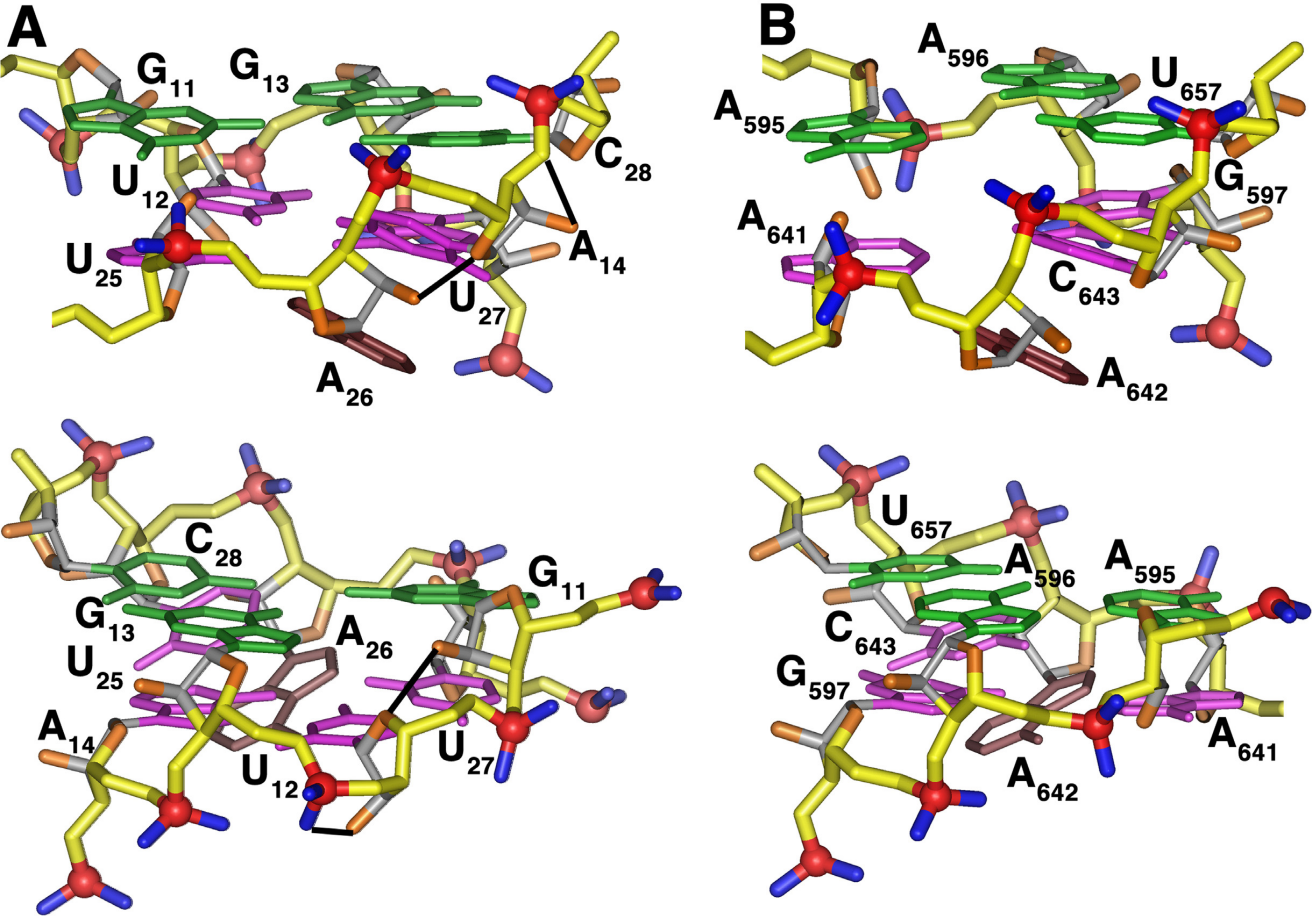
Comparison of the non-canonical regions of **(A)** the aptamer RNA aptamer in complex with *Bacillus* r-protein S8 and **(B)** the *spc* mRNA in complex with *E. coli* r-protein. The structurally homologous base triples and adenine base are shown in green and brown, respectively. Intra-molecular hydrogen bonds unique to the aptamer (A) are depicted in black. The phosphate groups that interact with the S8 proteins have a very similar distribution (upper). The phosphate group of the additional residue in the core region of the aptamer is accommodated on the phosphate backbone strand distal to the protein surface (lower).

The structure of aptamer RNA-2 is well defined with a global fold of a hairpin terminated on one end by the UUCG tetraloop. The tetraloop nucleotides adopt the archetypal conformation with U1 and G4 of the loop pairing and G4 adopting the *syn* configuration about the glycosidic bond. The canonical A-form helical stem of the aptamer is interrupted by an internal loop that includes nucleotides A_10_-A_14_ on the 5′ strand and U_25_-A_29_ on the 3′ strand. This central core of the aptamer has several non-standard structure features and is characterized by a complex network hydrogen bonds among the bases. A_10_ and A_29_ at one end of the loop adopt a *cis* Watson–Crick/Watson–Crick base pair via an A_10_ N1-A_29_ NH_2_ hydrogen bond and weak A_10_ H2-A_29_ N1 hydrogen bond (similar to that between A_1912_-A_1927_ in *Haloarcula marismortui* 23S rRNA). Stacked against the A_10_-A_29_ pair is a G_11_-(G_13_-C_28_) base triple. The base of G_11_ is coplanar with the Watson–Crick G_13_-C_28_ base pair and is joined to the pair through hydrogen bond G_13_ O6-G_11_ NH_2_. The G_11_ base is further locked into position by a hydrogen bond between G_11_ O6 and U_25_ 2′-OH. This base triple stacks on a base quartet composed of residues U_12_, A_14_, U_25_ and U_27_ (Figure 4). A_14_ and U_27_ form a buckled Watson–Crick A-U base pair. The U_12_ N3H and O4 atoms form hydrogen bonds with A_14_ N7 and N6H_2_, respectively. Residue U_25_ hydrogen bonds with both U_12_ (U_25_ N3H to U_12_ O4) and A_14_ (U_25_ O2 to A_14_ NH_2_) and is coplanar with A_14_ and U_25_ (Figure 4). The base of A_26_ stacks beneath U_27_ and is positioned by hydrogen bonds between A_26_ NH_2_ and U_15_ and U_25_ O2 atoms. A_26_ is displaced to the minor groove side of the helix axis and terminates the base stack along the 3′ strand of the stem. This arrangement of nucleotides flattens the pitch of the 5′ strand of the phosphate backbone through the internal loop of the aptamer. In contrast, the 3′ strand of the phosphate backbone maintains its pitch through the internal loop. In particular, the leapfrog effect of the U_12_ and G_13_ bases that occupy adjacent planes and the displaced A_26_ nucleotide alters the register of the phosphate groups along the 5′ and 3′ strands of the stem, respectively (Figure 5).

**Figure 6.**
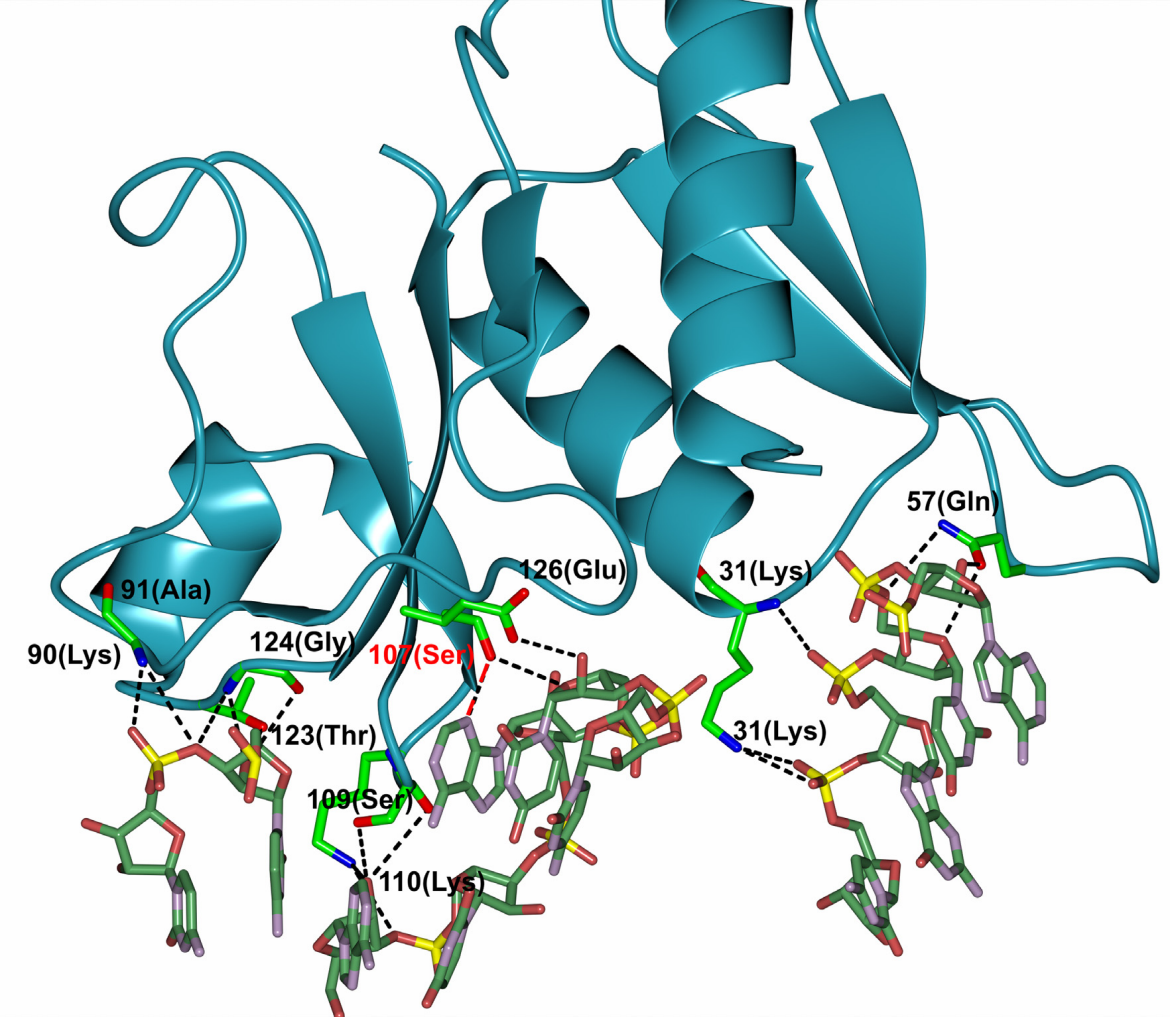
Intermolecular hydrogen-bond and electrostatic interactions between r-protein S8 and the RNA aptamer. The single direct base contact, S107 NH-A26 N3, is highlighted in red.

The interaction between r-protein S8 and the RNA aptamer involves one face of the RNA and extends from base pairs A_4_-U_35_ to C_17_-G_22_. This interaction buries approximately 923 Å^2^ of protein surface area which is similar to the 870 Å^2^ and 940 Å^2^ reported for the *E. coli* S8-*spc* mRNA and *Methanococcus jannaschii* S8-rRNA complexes, respectively (19,20). There are about twice as many protein–RNA contacts arising from the C-terminal domain of r-protein S8 than from the N-terminal domain and include electrostatic, hydrogen-bond and hydrophobic interactions. All but one of the protein–RNA contacts involve the sugar-phosphate backbone; the only base interaction is between A_26_ N3 and the side chain hydroxyl of S107 (Figure 6). The side chain of K31 forms a salt bridge with the pC_2_ phosphate and the backbone amide forms a hydrogen bond with pG_1_ phosphate. The side chains of T4 and Q57 interact with pA_4_ and the A_4_ 2′ OH and the side chain of K56 forms a hydrogen bond with the C_36_ 2′ OH.

The interface between the RNA and the C-terminal domain of S8 includes specific interactions in the core of the RNA and non-specific interactions with the sugar-phosphate backbone of the internal loop and stem. The tetraloop nucleotides do not interact with the protein. The phosphoryl oxygens and 2′ OH groups of C_16_, C_17_, A_24_, U_25_, U_27_ and A_26_ form salt bridges or hydrogen bonds with side chain or backbone amide functional groups of E126, S107, G124, K110, S109, A91 and T123 (Figure 6). In addition to forming the only base-specific contact, the side chain of S107 also forms a hydrogen bond with the A_26_ 2′ OH. Additional protein–RNA interactions in the complex are mediated by water molecules and include base contacts to internal loop residues U_27_ O2 to E126 OE1 and C_28_ O2 to Y88 OH. Also, the peptide bond between the highly conserved residues S107-T108-S109 stacks against the purine ring of A_26_. An analogous stacking interaction is present in the complex between r-protein S8 and its natural RNA targets and involves A_642_ (20,21).

## DISCUSSION

Ribosomal protein S8 is highly conserved among bacteria and archaea and serves as a translational repressor of ribosomal protein genes in the bacterial *spc* operon (15). The contacts between S8 and its RNA targets are largely the same within the contexts of the *spc* mRNA (19), helix 21 of 16S rRNA (20) and the 30S ribosomal subunit (21). Many of the native-like contacts also are present in the S8-aptamer complex, but the primary structure of the aptamer requires a novel network of nucleobase interactions to form the complex.

### Comparison of the aptamer structures in the free and S8-bound states

**Figure 7.**
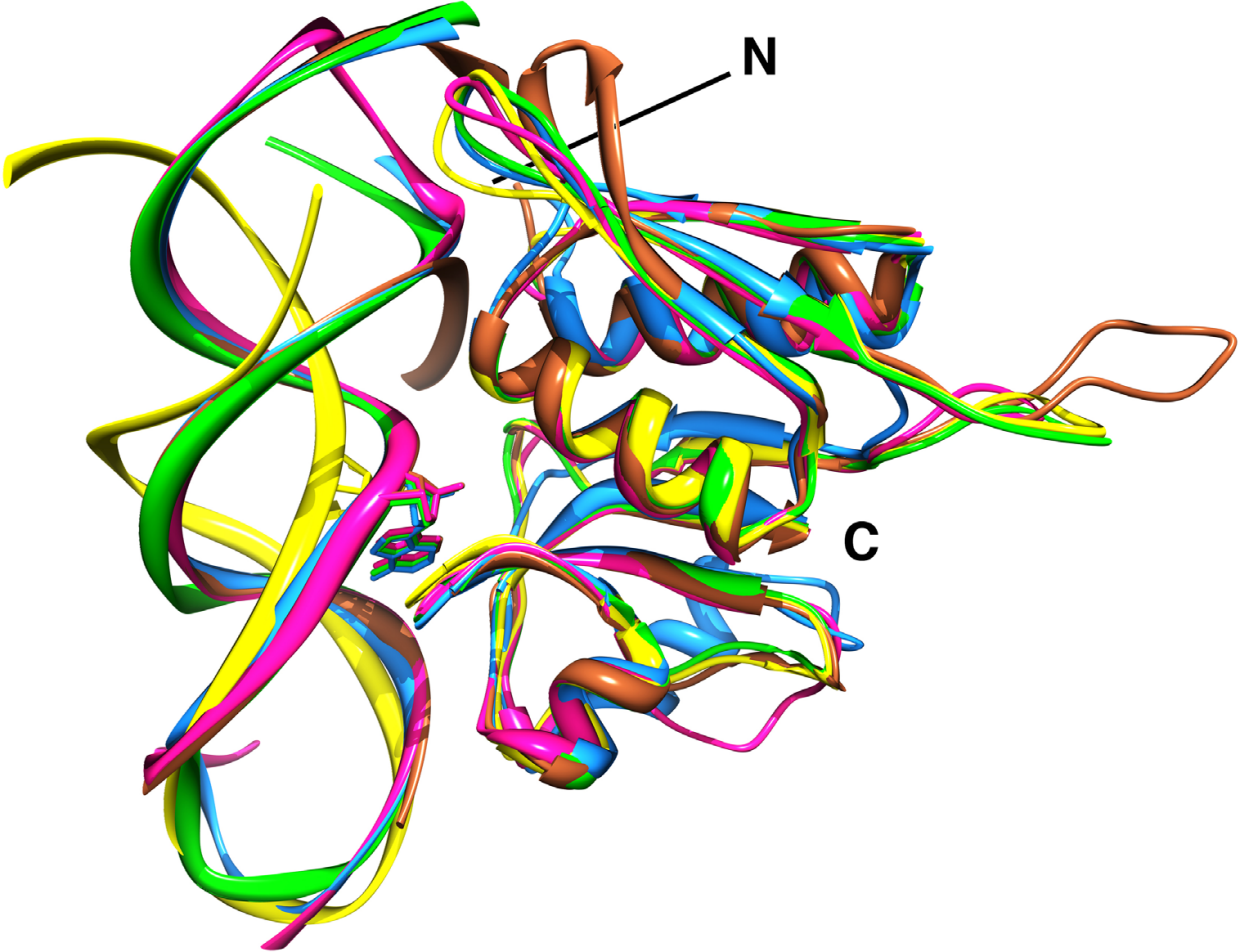
Superposition of peptide backbone atoms of S8-RNA complexes from *Bacillus* S8 (green) with those of *E. coli* S8 (PDBID 1S03) (magenta), *M. jannaschii* S8 (PDBID 1I6U) (blue) and *Thermus thermophilus* S8 (PDBID 1FJF) (brown). The backbone of the RNA-free *Bacillus* S8 (this study) and the solution structure of RNA-1 are shown in yellow. The highly conserved adenine (A_642_*E. coli* 16S rRNA) of the RNA binding site lies above the similarly conserved S-T-S/T (105–106–107 *E. coli* S8) tripeptide to form a π–π stacking interaction. *T. thermophilus* S8 (brown) contains an extended loop between N- and C- terminal domains that forms additional interactions with 16S rRNA, whereas the corresponding loop in *M. janasschii* (blue) is truncated.

**Figure 8.**
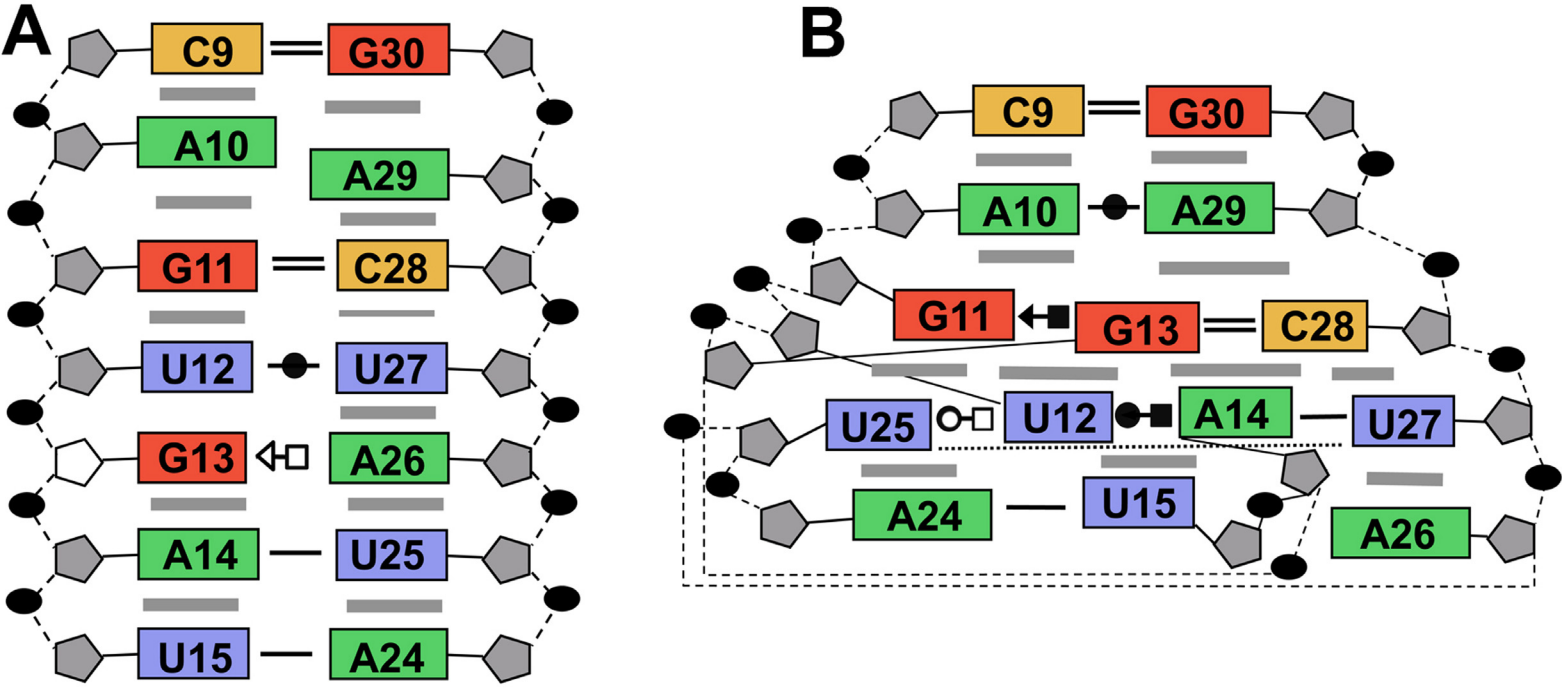
Comparison of stacking and hydrogen-bond interactions for **(A)** free and **(B)** S8-bound forms of the internal loop of the aptamer. Gray bars indicate base stacking and base–base interactions are indicated using the geometric nomenclature as described (45). The ribose of G_13_ adopts the C2′-*endo* ring pucker in the free RNA.

The structure of the protein-free RNA aptamer in solution is well ordered and exhibits negligible intermediate time-scale dynamics. Nucleotides G_11_-A_14_ and U_25_-C_28_ form the central portion of the stem and core binding site for r-protein S8. The non-canonical U_12_-U_27_ and G_13_-A_26_ base pairs are somewhat relaxed from idealized geometries and the purine rings of the A_10_-A_29_ mismatch lie on overlapping planes, leading to a small kink in the helix. The G-C and A-U base pairs that flank the internal loop exhibit increased solvent accessibility as evidenced by rapid NH solvent exchange. The conformation of the RNA binding site core region is substantially altered in the complex (Figure 7). The G_13_-A_26_ pair is disrupted as the base of G_13_ leapfrogs over U_12_ to pair with C_28_ and forms a base triple via the minor groove edge of G_11_ (Figures 4 and 8) (45). The base of A_26_ is displaced from the helix but continues to stack beneath the plane the adjacent U_27_ residue. The U_12_-U_27_ and A_14_-U_25_ base pairs are remolded into a base quartet tethered together by an array of hydrogen bonds largely involving functional groups of the major groove base edges (Figure 5) (45). Residue A_14_ continues to participate in a Watson–Crick-type base pair, but its pairing partner changes from U_25_ to U_27_. This arrangement of core nucleotide interactions appears unique among free or RNA-ligand complexes (46).

### Comparison of the S8-aptamer complex with native S8-RNA complexes

Despite the obvious sequence and structural differences between the native RNA sites and the aptamer, the structure of the aptamer is dramatically remodeled in the S8 complex to produce a conformation with remarkable similarities to native S8-RNA complexes (19–21). Alignment of residues and superposition of peptide backbone atoms from *Bacillus* S8 with those of *E. coli* S8 (21), *M. jannaschii* S8 (20) and *A. aeolicus* S8 (17) result in rmsds of 0.57 Å, 0.78 Å and 0.65 Å, respectively (Figure 7). Also, many of the inter-molecular interactions common to the native S8-RNA complexes, which are generally well conserved, are recapitulated in the RNA aptamer-S8 complex. Shape complementarity, electrostatic and hydrogen-bond interactions are key features of the S8-RNA interaction. The invariant A_642_ in helix 21 participates in the only conserved base-specific contact, a hydrogen bond between the conserved serine 106 side chain (*E. coli* numbering) and the A_642_ N3 atom. A_26_ functionally replaces A_642_ in the S8-aptamer complex (Figure 6). The only other base contact in some of the natural S8-RNA complexes is a hydrogen bond between the G_597_ N2 and the Y85 side chain OH. In the archaeon *M. jannaschii*, R124 is positioned at the site that Y85 occupies in *E. coli* (and other eubacterial S8 proteins). The R124 side chain NH_2_ interacts with the G_597_ N3 and U_598_ O4′. In the aptamer-S8 complex, an interaction analogous to Y85-G_597_ involving the G_11_-(G_13_-C_28_) base triple is not observed. Many of the other interactions present in the S8-RNA structures correlate with earlier biochemical analyses (11–13,47,48). One notable exception is the hydrogen bond from the S107 side chain to the A_640_ 2′-OH present in native complexes. Substitution with deoxy-A at 640 does not affect protein binding (48). This interaction also is present in the S8-aptamer complex between the homologous S109 and A_24_ (which is isomorphic with A_640_).

The RNA selection was designed to identify alternative modes that the S8 protein could use to bind RNA. We expected the topology of the S8 binding site on helix 21 to be incompatible with a symmetric internal loop, but the S8-RNA interface is remarkably well preserved. In addition, a critical π–π stacking interaction involving the purine ring of A_642_ and the T106-S107 (*E. coli* S8) peptide bond is recapitulated. This interaction is facilitated in 16S rRNA and *spc* mRNA by the odd number of nucleotides in the internal loop. In the aptamer, the stacking of the A_26_ base is made possible when the G_13_ and A_26_ bases shift above and below the plane of the base quartet, respectively. In the natural RNA targets, A_642_ participates in an *i* to *i*+1 base pair with residue 641 (20,21,23), and U_25_ and A_26_ form a similar hydrogen bond. Rotamer analysis reveals the phosphate backbones at steps U_25_-A_26_ of the aptamer and N_641_-A_642_ of the natural RNA sites have the same geometry and that it is characteristic of *i* to *i*+1 base pairs (49). The base triple is another feature common to the aptmer and natural RNA binding sites. In *E. coli* helix 21, the triple is A_595_-(A_596_-U_644_) and in *T. thermophilus*, G_595_-(C_596_-G_644_). In the *spc* mRNA binding site, the corresponding base triple is A_+80_-(A_+81_-U_+11_). Although the base triples are isosteric, the non-Watson–Crick residue of the base triple in the aptamer RNA, G_11_-(G_13_-C_28_), is not sequential with either nucleotide of the base pair. This nucleotide topology difference for the aptamer base triple is reflected in the local geometry of the backbone on the face opposite the bound S8 protein. The backbone geometry at the G_11_-U_12_ step is characteristic of the loop E motif (49), and although the corresponding positions of natural RNA sites are non-A-like, they do not have the loop E motif geometry (Figure 5). Thus, backbone perturbations caused by the symmetric internal loop of the aptamer are contained to the RNA face opposite the bound S8 protein (Figure 5).

### Implications for aptamer–protein structure and interaction

Two sites on r-protein S8 interact with 16S rRNA, one site involves helix 21 and a second site involves helix 25. Therefore, S8 presents at least two surfaces for an RNA aptamer. The helix 21 binding site on S8 is lined with a strip of electropositive charge along which the phosphate backbone of the aptamer traverses from residues A_4_-C_7_ and U_27_-A_29_ (Supplementary Figure S3). Nodes of electropositive density also are centered at residues C_17_ and U_25_, but an electronegative patch in this primary binding site contours to the minor groove edge of A_26_. The electropositive surface charge that lines the secondary RNA binding site on S8 is more extensive than that on the primary face, but RNA binding in this region is weaker and non-specific. Given the potential for multiple charge–charge interactions, it is somewhat surprising that the secondary binding site was not identified as a preferred target during the selection. However, a site that accommodates multiple types of interactions (electrostatic, hydrogen bond, van der Waals) might be favored since electrostatic contributions toward binding diminish due to shielding effects caused by increasing salt concentrations used during the selection.

Protein surfaces present specific structured sites, or epitopes, that are recognized by aptamers and often the same protein epitope can bind aptamers of different sequence and potentially different structure (50–52). The characterization of most aptamer–protein interactions has been limited to affinity or kinetic measurements with few high-resolution structures of aptamer–protein complexes reported (53) and only four complexes involving nucleic acid binding proteins (52,54–56). Thus, although a protein epitope can bind aptamers from different sequence (and potentially of different structure) classes, the extent of similarity among the binding modes, the conservation of intermolecular interactions and the structural heterogeneity of the aptamers must largely be inferred.

Three complexes that offer a basis for comparison of free and bound aptamers as well as comparison of binding modes of aptamer and natural targets involve the MS2 capsid protein, NF-κB p50 homodimer and nucleolin. Aptamers against the MS2 capsid protein have the same basic secondary structure as the natural RNA binding site, an RNA hairpin capped by a four-nucleotide loop, and form many of the same protein–RNA interactions (52). One class of aptamer, though, adopts a hairpin that contains a *three*-nucleotide loop, yet forms many of the same interactions with capsid protein as the natural RNA ligand. In the case of the NF-κB p50 homodimer, the RNA aptamer forms a hairpin with a seven-nucleotide internal loop capped by a GNRA tetraloop (57). In the complex, the aptamer binds to each monomer of the dimer and forms several base-specific protein contacts. The structure differences between free and bound aptamer are small but include altered base stacking in the tetraloop and stabilization of a U-C base pair in the internal loop (54,57). Thus, for the MS2 capsid protein and the NF-κB dimer, not only do the natural nucleic acid binding sites serve as epitopes, but the aptamers bind the core regions using the same chemistry as the natural ligands. In addition, the conformations of the free and bound states of the aptamers are well ordered and exhibit few differences. In the case of nucleolin, protein–RNA interactions that comprise a natural RNA ligand:nucleolin complex are a subset of the interactions present in the aptamer:nucleolin complex (58). Nucleotides that are not conserved within the natural RNA targets or that are not part of the consensus sequence of the aptamer RNA become ordered only upon protein binding (56,58).

As with the NF-κB and MS2 capsid protein complexes, the interactions between the aptamer and S8 recapitulate those of the native complexes. However, only the S8 aptamer has significant structural dissimilarity between free and protein-bound forms (Figure 8). The secondary structure properties of the aptamer also contrast those of the natural targets of S8 which are the same in free and bound states (20,21,23,24). RNA aptamers against proteins that do not naturally bind nucleic acids also are found to adopt the bound conformation in the free state (59–63). The S8 apatmer is the first example of an RNA aptamer that adopts substantially different secondary structures in the free and protein-bound states. It is possible that the bound conformation of the S8 aptamer also is present in solution, albeit at very low abundance and in rapid exchange with the duplex conformation, which could be captured by r-protein S8. Although the number of examples is limited, the breaking and reorganization of multiple secondary structure elements within an RNA aptamer upon protein binding appears to be uncommon.

## ACCESSION NUMBERS

Coordinates have been deposited in the Protein Data Bank under accession numbers PDB ID: 2LUN, solution structure of the RNA aptamer, and 4PDB, crystal structure of the S8-aptamer complex. Chemical shifts have been deposited in the Biomolecular Magnetic Resonance Bank under accession numbers BMRB ID: 18532.

## SUPPLEMENTARY DATA

Supplementary Data are available at NAR Online.

SUPPLEMENTARY DATA
