# Supplementary material for: Structure analysis of free and bound states of an RNA aptamer against ribosomal protein S8 from Bacillus anthracis
Source: Nucleic Acids Res. 2014 Aug 19;42(16):10795–808. doi: 10.1093/nar/gku743 (PMC4176348; doi:10.1093/nar/gku743)
Supplement: SUPPLEMENTARY DATA [file supp_42_16_10795__index.html]

Structure analysis of free and bound states of an RNA aptamer against ribosomal protein S8 from Bacillus anthracis — SUPPLEMENTARY DATA 

# Structure analysis of free and bound states of an RNA aptamer against ribosomal protein S8 from *Bacillus anthracis*

## SUPPLEMENTARY DATA

**Files in this Data Supplement:**

- SUPPLEMENTARY DATA
