# Supplementary material for: Structure analysis of free and bound states of an RNA aptamer against ribosomal protein S8 from Bacillus anthracis
Source: Nucleic Acids Res. 2014 Aug 19;42(16):10795–808. doi: 10.1093/nar/gku743 (PMC4176348; doi:10.1093/nar/gku743)
Supplement: SUPPLEMENTARY DATA [file supp_gku743_nar-01257-r-2014-File010.pdf]

Supporting Information

Structure Analysis of free and bound states of an RNA Aptamer Against Ribosomal Protein S8 from *Bacillus anthracis* †

Milya Davlieva[1], James Donarski[2], Jiachen Wang[3], Yousif Shamoo[1] and Edward P. Nikonowicz[1,*]

[1]Department of Biochemistry and Cell Biology Rice University, Houston, TX 77251-1892

[2]Food and Environment Research Agency, Sand Hutton, York, YO41 1LZ, United Kingdom

[3]Department of Physics, East China Normal University, 200062 Shanghai, P. R. China

Methods

In vitro RNA selection. The RNA aptamer selection experiment was performed as described (1-3) using the template 5'-TAATACGACTCACTATAGGGATCGAGGCTTCCT(N_Y)CUUCGG(N_X) GGGAAGCCTCCAAGCT-3' where the T7 promoter sequence is underlined. X and Y designate the number of randomized nucleotides (X=7, 8, 9 and Y=9, 8, 7) for a total of 16 randomized nucleotides. The primer for the reverse transcription reaction was 5'-GGGGACGAAGCTTGGAGGCTTCCC-3'. The template was purified via denaturing PAGE, electroeluted, and amplified via PCR. A 10 ml transcription reaction was performed using the template and T7 RNA polymerase (4) and the resulting RNA pool isolated via denaturing PAGE, electroeluted (Schleicher & Schuell), and precipitated from ethanol. The RNA was suspended in binding buffer (10 mM Tris-HCl, pH 7.5, 1 mM DTT, 50 mM KCl, and 20 mM $MgCl_2$) and the RNA pool passed through a nitrocellulose filter (Millipore) to remove RNA molecules that bound the filter. The unbound RNA was precipitated from ethanol with tRNA carrier, reverse transcribed, the template amplified by PCR followed by transcription to generate the preselected

RNA pool. The preselected RNA pool was incubated with *B. anthracis* r-protein S8 (2.0 μM) for 30 min at 4 °C and the mixture passed through a nitrocellulose filter and the filter washed with binding buffer. The S8-bound RNA was eluted from the filters using binding buffer with 0.8 M urea and 0.5 M NaCl, precipitated from ethanol, reverse transcribed into cDNA, and PCR amplified to complete the first round of SELEX. For the next 4 rounds of selection, the KCl concentration of the binding buffer was increased: 50, 100, 200, and 400 mM. For the next 4 rounds of selection, 400 mM KCl was used and the concentrations of S8 protein in the binding reactions were sequentially reduced by 5-fold. A tenth round of selection was repeated using the last, most stringent set of conditions. DNA from the final round was cloned into the pGEM-T plasmid (Promega) and after transformation, 50 independent clones were isolated and the plasmid inserts sequenced.

References

1. Conrad, R.C., Giver, L., Tian, Y. and Ellington, A.D. (1996) In vitro selection of nucleic acid aptamers that bind proteins. *Methods in enzymology*, **267**, 336-367.
2. Kenan, D.J. and Keene, J.D. (1999) In vitro selection of aptamers from RNA libraries. *Methods in molecular biology (Clifton, N.J*, **118**, 217-231.
3. Wilson, D.S. and Szostak, J.W. (1999) In vitro selection of functional nucleic acids. *Annual review of biochemistry*, **68**, 611-647.
4. Milligan, J.F., Groebe, D.R., Witherell, G.W. and Uhlenbeck, O.C. (1987) Oligoribonucleotide synthesis using T7 RNA Polymerase and Synthetic DNA Templates. *Nucleic Acids Research*, **15**, 8783-8789.

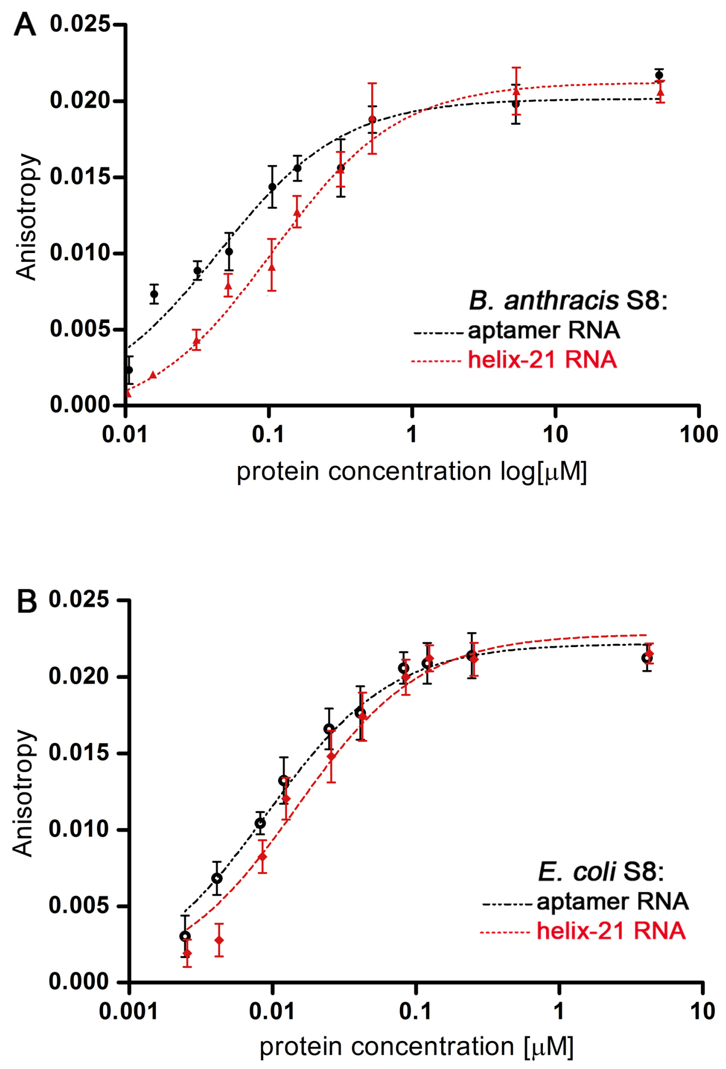

Figure S1. Plots of fluorescence polarization of fluorescein labeled RNA as a function of log r-protein S8 concentration. Samples were allowed to incubate at 4 °C for 5 minutes after mixing before fluorescence measurement. *B. anthracis* (A) and *E. coli* (B) r-protein S8 titrated with aptamer RNA (black) and helix-21 RNA (red).

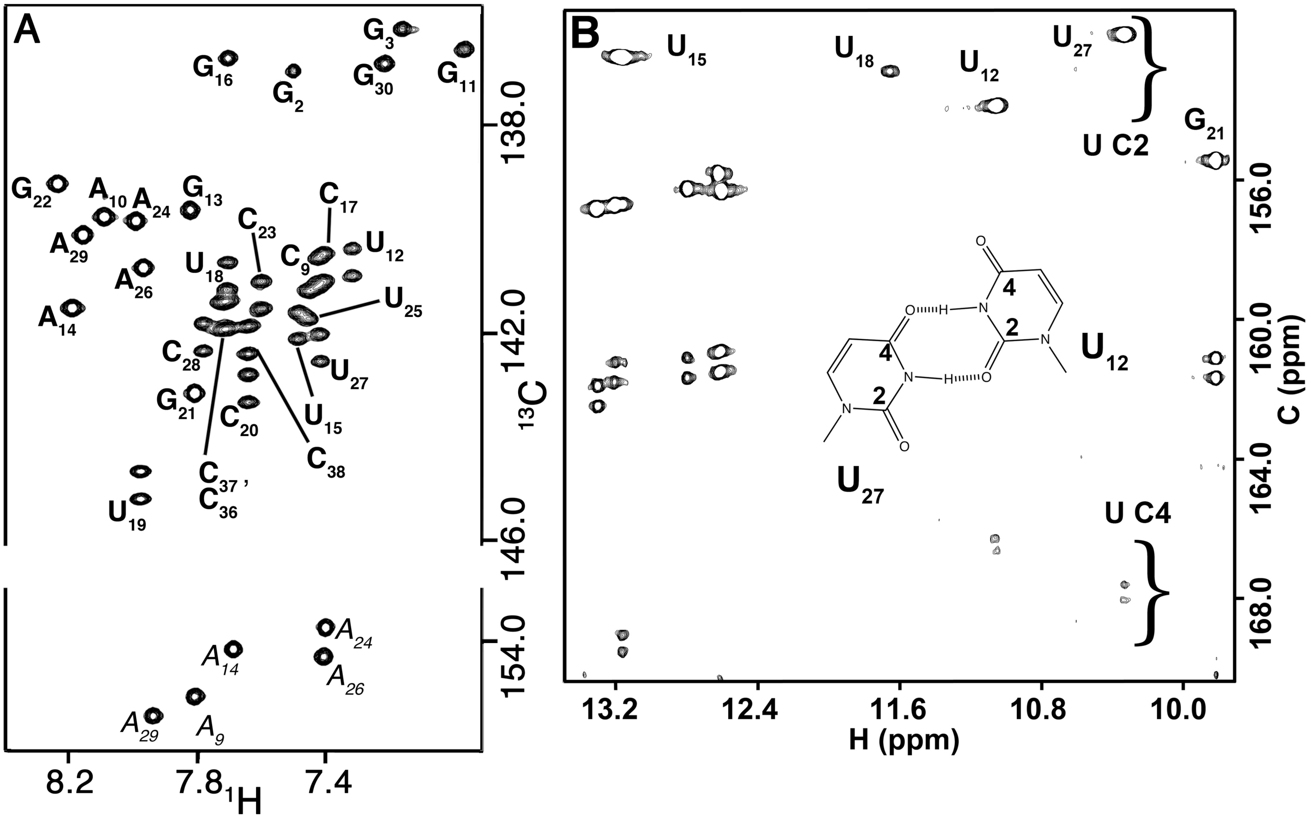

Figure S2. (A) Base region of 2D ^{1}H-^{13}C HSQC of RNA-1. (B) Imino H1/3-base carbon region of 2D H(N)CO spectrum of RNA-1 used to determine hydrogen bond scheme of the U_{12}-U_{27} base pair. The H(N)CO experiment was optimized to obtain intra-residue correlations. U_{12} and U_{27} imino resonances were assigned through C2 and C4 chemical shifts of the 2D H(CN)C spectrum. The hydrogen bond scheme was determined based on anomalous ^{13}C chemical shift values and the internal A-U and tetraloop U C2 and C4 chemical shift values.

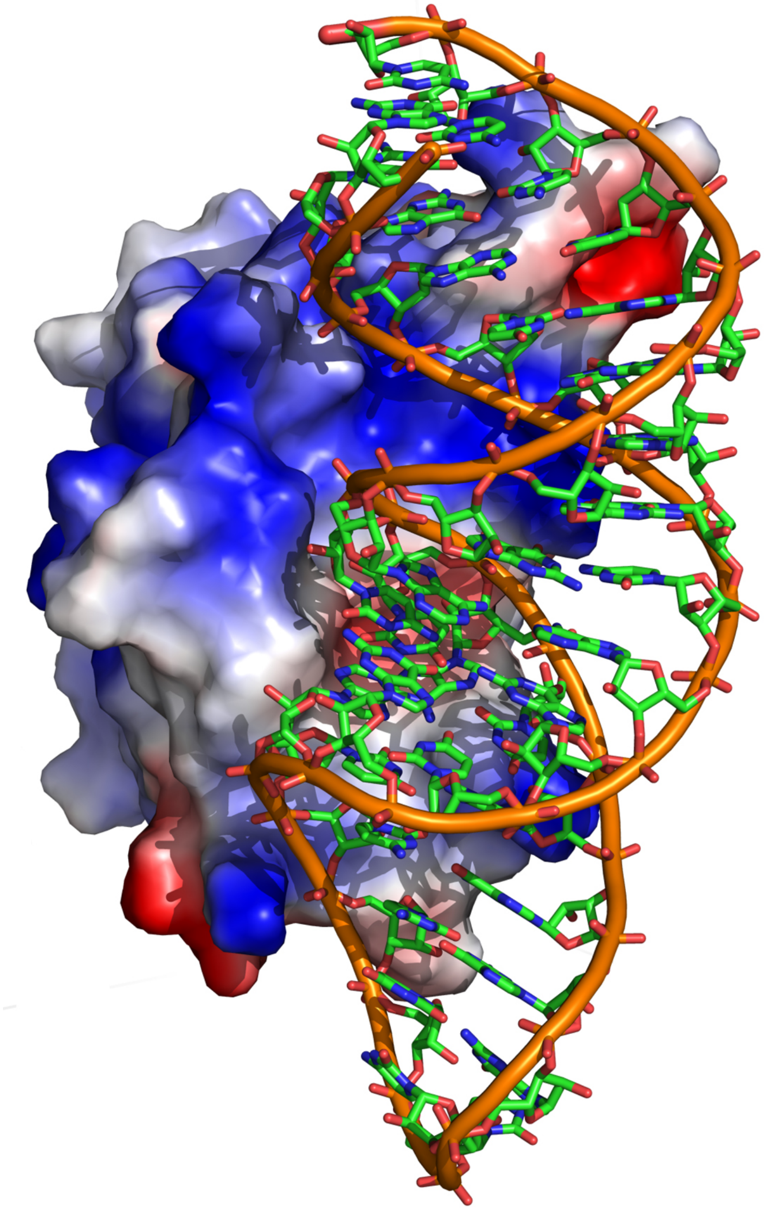

Figure S3. Rendering of the expected surface charge of S8 and the RNA aptamer. A large electropositive patch (blue) on the S8 surface complements the phosphate backbone of the aptamer formed by the Watson-Crick stem distal to the tetraloop. The intermolecular base and ribose hydrogen bonds and π-stacking interactions involving the non-canonical region of the RNA aptamer originate from a smaller electronegative patch (red) on the S8 protein.

Table S1. NMR experimental constraint and refinement statistics for the RNA apatmer.

**Constraint**		
**NOE distance constraints**		
total (including intra-ribose)		524
[a]intra-residue		152
inter-residue		148
[a]mean number per residue		10.7
**NOE constraints by category**		
very strong	(0.0 - 3.0 Å)	15
strong	(0.0 - 4.0 Å)	66
medium	(0.0 - 5.0 Å)	92
weak	(0.0 - 6.0 Å)	90
very weak	(0.0 - 7.0 Å)	37
**base pair constraints**		
total		62
**dihedral angle constraints**		
[b]ribose ring		78
backbone		115
mean number per residue		6.9
**violations**		
average distance constraints > 0.3Å		0.22
average dihedral constraints > 0.5˚		1.8
[c]**RMSD from ideal geometry**		
bonds (Å)		0.003
angles (˚)		0.72

[a]Only conformationally restrictive constraints are included, intra-ribose constraints are not included.

[b]Three torsion angles within each ribose ring were used to constrain the ring to either the C2'-*endo* or C3'-*endo* conformation.

[c]Calculated for the minimized average structure.

Table S2. Data collection and refinement statistics for S8-RNA complex.

**Data collection**	
Wavelength (Å)	1.5418
Resolution (Å)[a]	28.21-2.60 (2.69-2.60)
Space group	P212121
Molecules per a.u.	1
Unit Cell (Å)	a = 55.41, b = 59.27, c = 92.25
	$\alpha = \beta = \gamma = 90.0°$
Unique reflections[a]	9796 (*958*)
Average redundancy[a]	7.08 (6.73)
Completeness (%)[a]	98.9 (99.9)
R_{merge} (%)[a,b]	9.2 (64.3)
Output <I/sigI>[a]	11.0 (2.7)
**Refinement**	
R_{work} (%)[c]	18.9
R_{free} (%)[d]	27.0
r.m.s.d.[e] from ideality	
Bonds (Å)	0.009
Angles (°)	1.28
Average B-factor ($Å^2$)	49.9
Ramachandran[f]	
favored (%)	90.6
disallowed (%)	0.0

[a] Values for the last shell are in parenthesis

[b] $R_{merge} = \Sigma | I - \langle I \rangle | / \Sigma I$, where I is measured intensity for reflections with indices of hkl.

[c] $R_{work} = \Sigma | F_o - F_c | / \Sigma | F_o |$ for all data with $F_o > 2\sigma(F_o)$ excluding data to calculate R_{free}

[d] $R_{free} = \Sigma | F_o - F_c | / \Sigma | F_o |$ for all data with $F_o > 2\sigma(F_o)$ excluded from refinement.

[e] Root mean square deviation

[f] Calculated by using MolProbity.

Table S3. ^{1}H, ^{13}C, ^{15}N, ^{31}P Chemical shift assignments.

residue	H6/H8	H2/H5	H1'	H2'	H3'	H4'	H5'/5''	NH
G1	8.05		5.81	4.87	4.56	4.36	4.02,3.90	
G2	7.50		5.89	4.78	4.47	4.52	4.42,4.17	12.78
G3	7.16		5.72	4.48	4.44	4.43	4.44,4.05	13.17
C9	7.43	5.14	5.40	4.37	4.48	4.34	4.49,4.04	
A10	8.09	7.82	5.99	4.64	4.60	4.44	4.45,4.13	
G11	6.96		5.39	4.53	4.52	4.39	4.24,3.96	n.o.
U12	7.36	5.07	5.38	4.06	4.39	4.23	4.27,4.00	11.08
G13	7.82		5.56	4.66	4.71	4.48	4.20,4.09	n.o.
A14	8.19	7.69	5.78	4.77	4.58	4.50	4.35,4.23	
U15	7.49	5.10	5.31	4.49	4.42	4.35	4.40,4.15	13.15
G16	7.70		5.79	4.44	4.28	4.41	4.30,4.12	12.60
C17	7.38	5.15	5.39	4.45	4.19	4.36	4.43,3.98	
U18	7.72	5.66	5.59	3.75	4.49	4.31	4.44,4.04/	11.68
U19	7.98	5.80	6.05	4.64	3.97	4.42	4.19,3.98	n.o.
C20	7.64	6.08	5.91	4.05	4.43	3.74	3.52,2.65	
G21	7.81		5.91	4.79	5.58	4.35	4.34,4.13	9.80
G22	8.24		4.39	4.40	4.22	4.33	4.43,4.21	13.29
C23	7.61	5.22	5.43	4.43	4.36	4.28	4.43,3.96	
A24	8.00	7.40	5.94	4.61	4.48	4.44		
U25	7.46	5.20	5.44	4.26	4.08	4.46		n.o.
A26	7.97	7.41	5.76	4.53	4.27	4.49	4.37,4.10	
U27	7.42	5.22	5.09	4.18	4.25	4.28	4.30,4.00	10.53
C28	7.78	5.66	5.49	4.33	4.52	4.30	4.37,4.04	
A29	8.16	7.94	6.04	4.65	4.61	4.50	4.43,4.16	
G30	7.21		5.57	4.50	4.25	4.45	4.29,4.05	12.47
C36	7.72	5.08	5.42	4.51	4.29	4.37	4.46,4.01	
C37	7.74	5.41	5.44	4.42	4.48	4.38	4.42,4.06	
C38	7.64	5.45	5.71	3.95	4.17	4.07	4.45,3.97	

The non-exchangeable ^{1}H chemical shifts were measured at 26 °C and pH 6.6 and are referenced with DSS set to 0.00 ppm. Exchangeable proton chemical shifts were measured at 12 °C. The uncertainties in the chemical shift values are ≈0.02 ppm. The 5' and 5'' protons are not stereospecifically assigned.

residue	C6/C8	C2/C5	C1’	C2’	C3’	C4’	C5’	N(H)
G1	138.97	-	-	74.11				
G2	136.91		92.12	75.24	73.08	82.28	66.29	148.23
G3	136.12		92.53	75.09	72.36	82.01	64.83	149.08
C9	140.81	96.96	93.39	75.40	72.27	81.92	64.32	
A10	139.74	155.45	91.29	76.21	73.70	82.19	65.57	
G11	136.52	103.06	92.43	75.02	74.06	82.64	67.81	n.o.
U12	140.74	103.65	92.79	75.61	72.63	82.99	64.68	158.44
G13	139.62	154.36	88.70	75.01	76.83	84.96	67.18	n.o.
A14	141.53	154.15	90.36	75.32	74.33	82.90	66.74	
U15	141.88	103.49	92.55	74.44	72.10			162.55
G16	136.66		91.83	75.09	73.17	82.19	66.83	148.10
C17	140.78	96.71	93.14	75.32	71.74	81.74	64.41	
U18	140.90	104.91	93.89	75.91	73.08	82.37	63.88	160.44
U19	144.94	105.32	88.39	74.30	77.72	86.83	67.81	n.o.
C20	143.07	98.09	88.24	77.55	80.22	84.33	67.22	
G21	143.16		93.95	77.45	75.76	83.08	68.97	143.65
G22	138.11		92.57	75.61	74.44	82.99	69.63	148.67
C23	141.30	96.69	92.47	75.32	71.92	82.10	64.14	
A24	139.78	153.75	92.36	75.84	72.69	n.a.	n.a.	
U25	141.48	103.46	91.76	77.33	70.40	n.a.	n.a.	n.o.
A26	140.74	154.31	91.70	75.40	73.97	83.17	66.82	
U27	142.32	102.99	92.84	74.79	72.63	82.46	64.95	157.20
C28	142.07	97.68	92.65	75.54	72.99	81.65	64.86	
A29	140.06	155.08	90.92	75.84	74.42	82.73	66.29	
G30	136.80		92.69	74.87	73.70	82.64	67.35	148.98
C36	141.65	96.07	93.27	75.17	71.74	81.83	64.23	
C37	141.70	97.12	93.76	75.84	73.34	82.37	65.42	
C38	142.14	97.66	92.16	77.40	69.86	83.35	65.40	

residue	YN1/ RN9	YC2/ GC2	YC4/ GC6	RN7	A N1	A N3	A/C N6/N4	NH_2	5’-P
G1	-	-	-	-	-	-	-		
G2	169.52			233.79					n.a.
G3	168.78			234.08					n.a.
C9	150.74	159.54	169.19				97.75	8.34,6.79	n.a.
A10	169.75			230.52	229.26	214.81	82.33	6.71	n.a.
G11	168.34			235.42					n.a.
U12	145.07	154.73	167.31						n.a.
G13	166.93			236.76					n.a.
A14	168.93			230.75	223.17	214.46	82.12	n.o.	n.a.
U15		153.30	169.58						n.a.
G16	169.82			234.83					-4.02
C17	151.61	159.42	169.24				99.29	8.44,6.79	n.a.
U18	147.12	153.78	168.48						-4.36
U19	144.02	155.84	169.55						-3.51
C20	150.77	160.81	168.74				93.63	7.06,6.20	-5.05
G21	171.75	156.38	162.35	231.71					-4.94
G22	170.41			233.12					-2.40
C23	152.02	159.56	169.27				97.49	8.55,6.75	-4.36
A24	170.93			231.19	222.20	213.82	83.56	n.o.	n.a.
U25	146.55	153.70	168.97						-3.51
A26	169.23			231.41	225.47	215.74	80.84	6.45	-5.05
U27	146.35	152.98	168.67						-4.94
C28	151.68	159.74	168.94				98.16	8.01,7.04	-2.40
A29	169.08			231.04	229.70	213.90	81.71	6.76	n.a.
G30	168.34			233.56					n.a.
C36	152.10	159.60	168.93				98.62	8.49,6.70	n.a.
C37	146.52	159.72	169.32				98.31	8.41,6.77	n.a.
C38	153.64	160.66	169.52				97.18	8.22,6.92	-4.61

The ^{13}C, ^{15}N, and ^{31}P frequencies corresponding to 0.00 ppm were set according to the 0.00 ppm ^{1}H (DSS) frequency at 26 °C using the ratio γ_C/γ_H , γ_P/γ_H, and γ_N/γ_H. The chemical shifts have uncertainties of ≈0.05 ppm and ≈0.03 ppm. Y = U or C; R = G or A.
